# Progressive pseudorheumatoid dysplasia involving a novel CCN6 mutation: a case report

**DOI:** 10.3389/fimmu.2024.1445420

**Published:** 2024-10-30

**Authors:** Yu Li, Zhengping Huang, Yun Yan, Feng Guo, Gang Wei, Yue Wang, Yu Xie

**Affiliations:** ^1^ Affiliated Hospital of Nanjing University of Chinese Medicine, Nanjing, Jiangsu, China; ^2^ Department of Rheumatology and Immunology, Guangdong Second Provincial General Hospital, Guangzhou, China; ^3^ Department of Rheumatology and Immunology, Affiliated Hospital of Nanjing University of Chinese Medicine, Nanjing, Jiangsu, China

**Keywords:** communication network factor 6 (CCN6) gene, case report, progressive pseudorheumatoid dysplasia (PPRD), c. 802T>C, c.624dup

## Abstract

This study aims to report a case of progressive pseudorheumatoid dysplasia (PPRD) with two kinds of cellular communication network factor 6 (CCN6) gene mutation. In this paper, the clinical profile and the process of diagnosis were analyzed, and the related literature was reviewed. A 15-year-old boy, who developed progressive ankle and hip joint pain and enlargement with spine involvement, was diagnosed with PPRD. The erythrocyte sedimentation rate and C-reactive protein (CRP) were in the normal range; rheumatoid factor and anti-cyclic citrullinated peptide antibody (ACPA) were all negative. Human leukocyte antigen 27 (HLA-B27) was also negative. Cene study discovered two kinds of mutations in CCN6 gene: c. 802T>C and c.624dup. Radiographic studies revealed spine platyspondyly and shaped beaked, osteoporosis, and bilateral symmetric bony enlargements of the interphalangeal joints. Hip shows bilateral acetabulum and femoral head bone marrow edema, which revealed hip arthritis. Gene detection, laboratory examination, and typical radiographic features are helpful for the diagnosis of PPRD. This is the first report of c. 802T>C and c.624dup mutations in patients with PPRD in our country.

## 1 Introduction

Progressive pseudorheumatoid dysplasia (PPRD) is a rare—autosomal recessive noninflammatory arthropathy. It is associated with WNT1-inducible signaling pathway 3 (WISP3) gene mutations, first reported by Wynne Davies (1), which the current name of this gene is now CCN6. As there is no diagnostic standard for it, according to the reported cases, it is characterized by progressive degeneration of articular cartilage that leads to a significant disability with pain, stiffness, and joint deformities (2). The patient’s physical signs are common finger joint and knee joint enlargement, an abnormal front-to-back curvature of the spine (kyphosis) and a short torso, normal level of serological inflammation (3). Imaging in a small number of cases reported showed inflammatory changes, joint space narrowing, and osteophyte formation. Common coracoid changes or platyspondyly of vertebral body. To date, more than 64 different CCN6 mutations have been reported in more than 200 PPRD patients (4).

PPRD is often misdiagnosed as juvenile rheumatoid arthritis (JIA), spondyloarthritis (SpA), and other inflammatory diseases clinically. In order to avoid misdiagnosis or delayed diagnosis, imaging and gene detection play an important role in the differential diagnosis of the two diseases. Herein, we describe a case of PPRD with rarely reported CCN6 mutations, c.802T>C and c.624dup. Additionally, the patient had bone marrow edema, which has not been previously reported.

## 2 Case presentation

**Figure 1:**
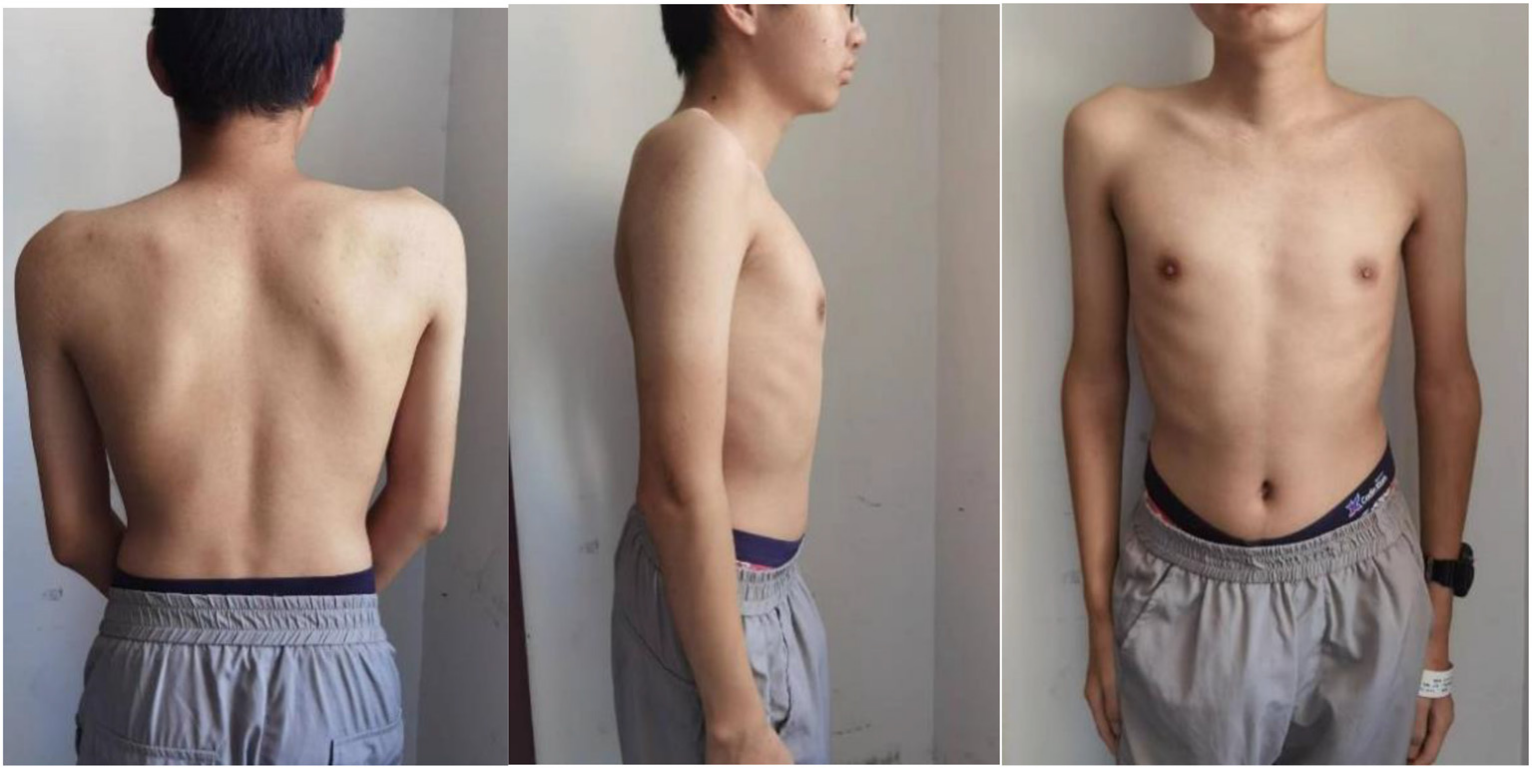
Obvious scoliosis with high and low shoulders, and he presented with scoliosis.

A 15-year-old boy (height: 1.62 m; weight: 47kg) was admitted with pain in ankle joints that had lasted for 5 years, who presented with scoliosis (**Figure 1**). In 2018, the patient experienced no apparent trigger for ankle joint pain and did not give it significant consideration. Until March 2019, when his ankle pain and swelling got worse so as to walk hard, he went to the hospital to check Biological markers, which contained complete blood count, ACPA, rheumatoid factor (RF), antinuclear antibody (ANA), extractable nuclear antigen (ENA), mlli—neutrophil cytoplasmic antibodies (ANCA), anti–double-stranded DNA antibody(anti–ds-DNA), enzyme-linked immunospot (ELISPOT), Hepatitis B virus-DNA (HBV-DNA), and Epstein-Barr virus-DNA (EB-DNA), and HLA-B27 quantification, which were all negative; and the antistreptolysin “O”(ASO) level was increased to 762 IU/L. Magnetic resonance imaging (MRI) revealed bilateral sacroiliac joint surface is not smooth with effusion, and multiple abnormal signals of bilateral heel talus and left foot scaphoid with calcaneal talus joint effusion. In order to make a clear diagnosis, the hospital completed bone marrow puncture that revealed granulocyte proliferation was active, erythroid and megakaryocytic proliferation was active, and platelet clusters were visible. The diagnosis made at the external hospital was JIA and streptococcal infection, for which the patient received intramuscular penicillin injections, disease-modifying antirheumatic drugs (DMARDs), nonsteroidal anti-inflammatory drugs (NSAIDs), calcium supplements, and stomach protection, which without any improvement so that he stopped the drug after 1 month. According to the patient’s past history, we are highly skeptical of his diagnosis of JIA, so that we focused on genetic testing and observations of the spine and hip joints. He did the biological markers and complete blood count (**Table 1**), and Radiological assessment after he was admitted to our hospital.

**Table 1:** Biological markers and complete blood count.

Parameter	Result	Reference range
Height	162 cm	
Weight	47 kg	
ANA	Negative	
ENA	Negative	
HLA-B27	Not detected	
Erythrocyte sedimentation rate (ESR)	9 mm/h	0–20 mm/h
CRP	5.45 mg/L	< 8 mg/L
RF	< 20 U/ml	< 20 U/ml
ASO	278.00	< 116.00 IU/ml
25 Determination of VD	11 ng/mL	> 20 ng/mL
Complete blood count		
White blood cell count	3.89 × 10^9/L	4.00–10.00 × 10^9/L
Hemoglobin	134 g/L	120–160 g/L
Neutrophils	41.5%	50.0%–70.0%
Lymphocytes	47.0%	20.0%–40.0%
Monocyte	9.5%	3.0%–8.0%

HLA B27, human leukocyte antigen B27; ANA, anti-nuclear antibody; RF, rheumatoid factor; ESR, erythrocyte sedimentation rate; CRP, C-reactive protein.

### 2.1 Radiological assessment

**Figure 2:**
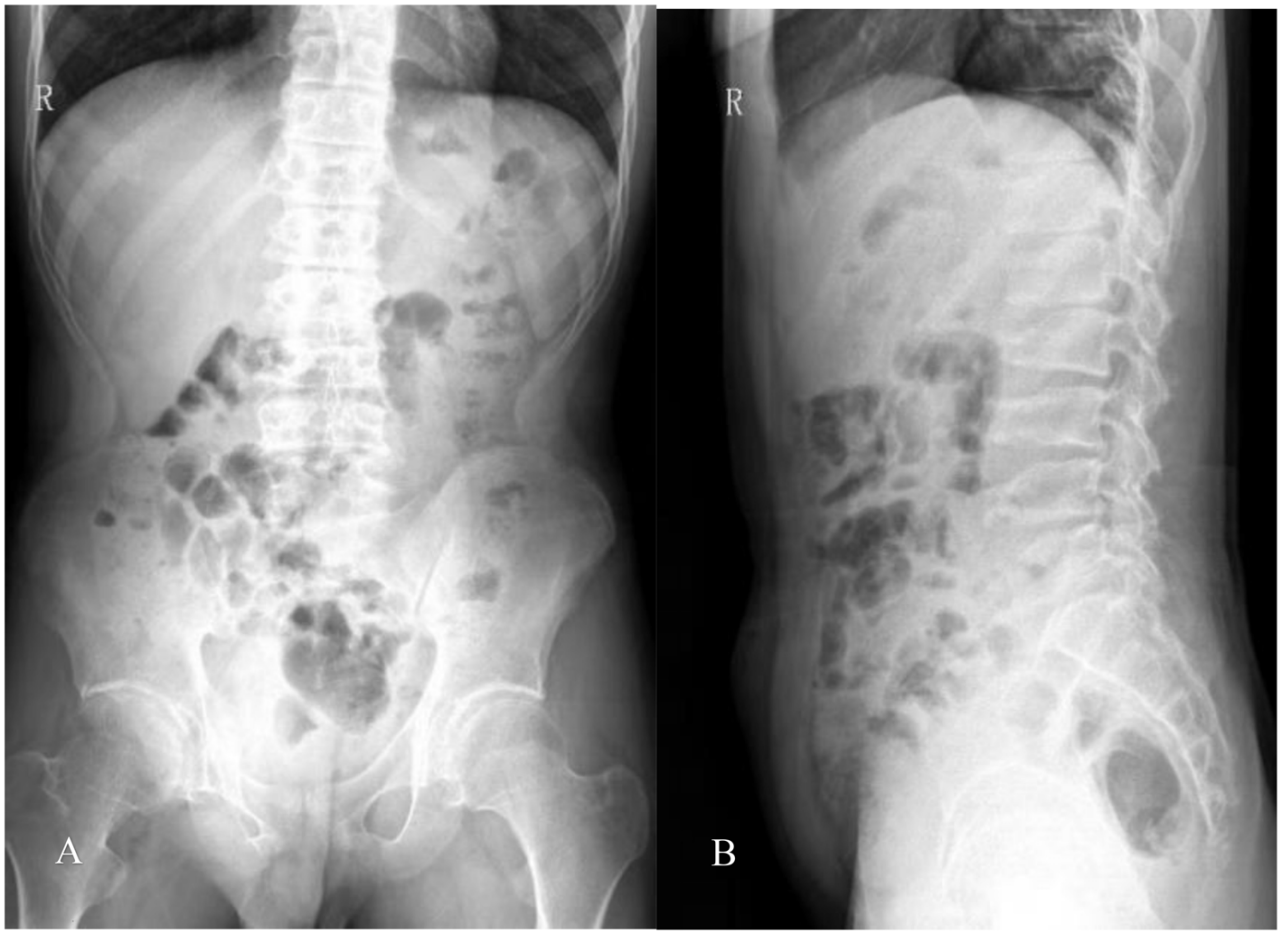
**(A)** Slight scoliosis of spine and narrowing of coxae space; **(B)** flat vertebra of cervical, thoracic, and lumbar segments, and the upper and lower edges of the vertebral body are concave, which showed coracoid changes; Irregular vertebral endplate and shortened pedicle.

**Figure 3:**
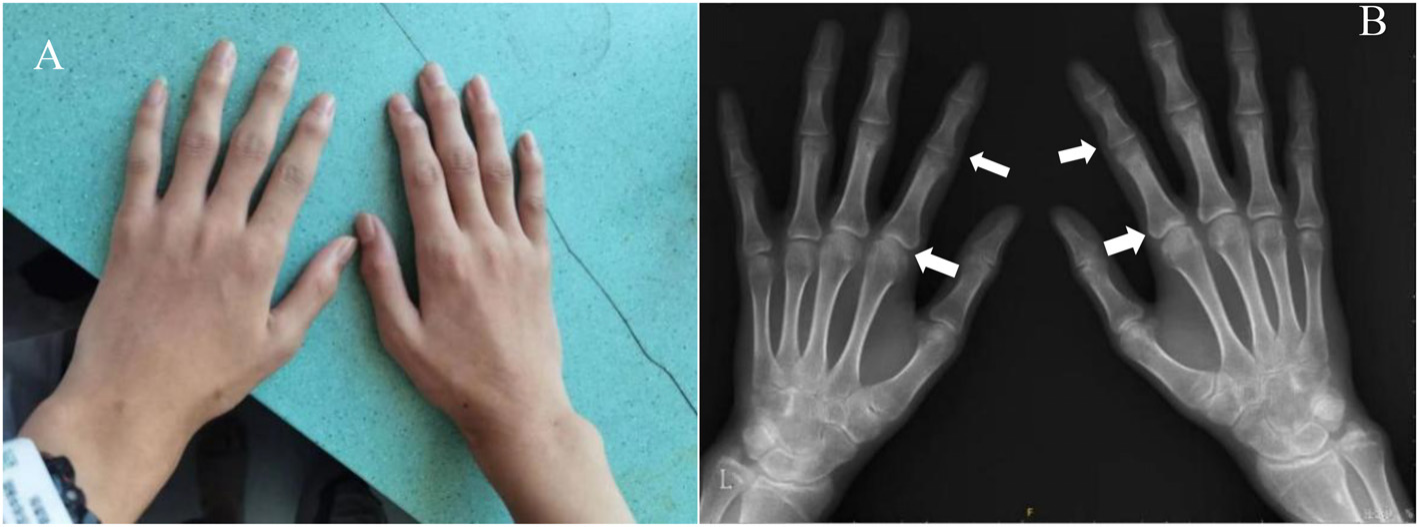
**(A)** Spindle-shaped enlargement of proximal and distal finger joints of both hands; **(B)** hands x-ray showing enlarged epiphyses and metaphyses of the metacarpals and phalanges, with joint spaces narrowing and osteophytic formations (arrows). The bone age of the patient was 16 years old. No significant inflammatory changes were observed.

**Figure 4:**
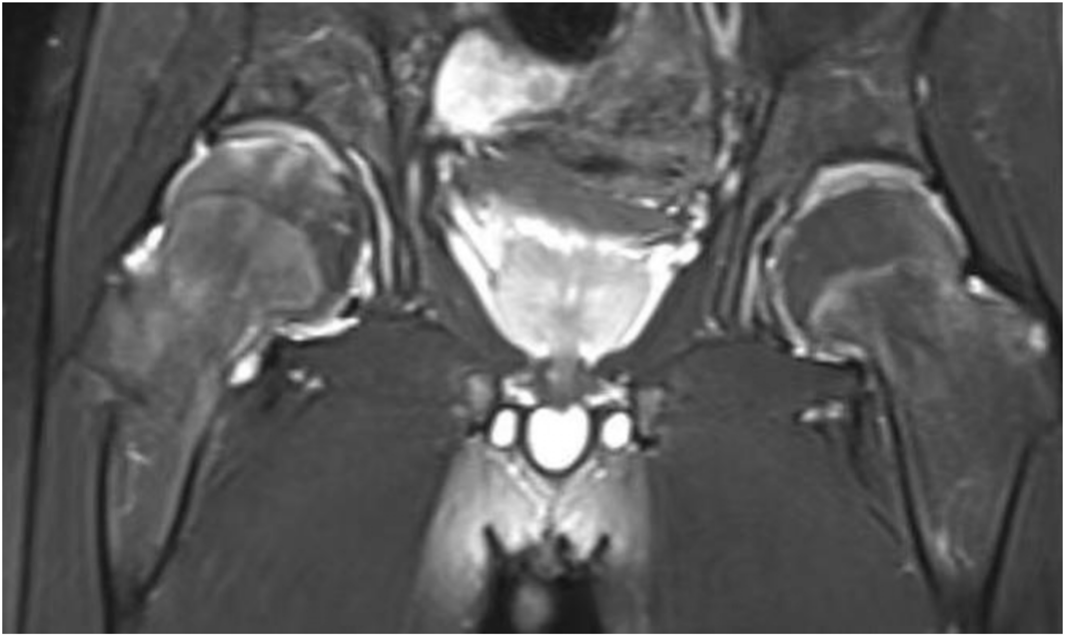
Hip shows bilateral acetabulum and femoral head bone marrow edema.

Radiography and computerized tomography (CT) showed no significant cardiopulmonary abnormalities. Radiography of spine showed abnormal changes in the shape of the lumbar spine presented platyspondyly and beaked which consistent with typical spinal manifestations of PPRD (**Figure 2B**). Spindle-shaped enlargement of proximal and distal finger joints of both hands (**Figure 3**). CT of sacroiliac joint revealed bilateral sacroiliac arthritis possibly. Coxae MRI revealed bilateral acetabulum and femoral head bone marrow edema (**Figure 4**), joint space narrowing (**Figure 2A**), and periarticular effusion which was similar to the imaging findings of ankylosing spondylitis. Spindle-shaped enlargement of proximal and distal finger joints of both hands.

**Figure 5:**
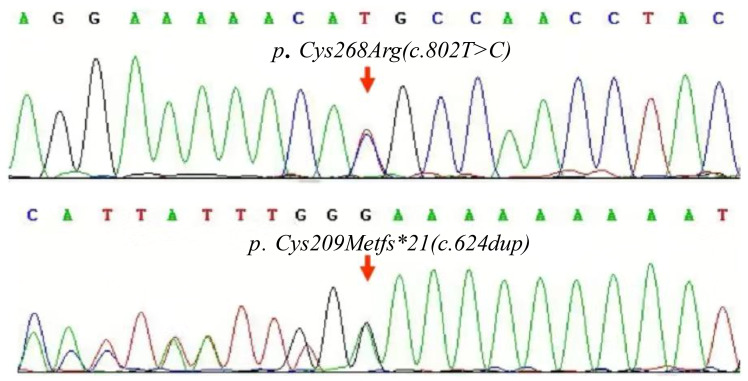
Maps of the CCN6 gene mutation in the patient. The compound heterozygous mutations, c.802T>C and c.624dup carried by the patient.

No abnormality was found in the inflammatory indicators of the patient, and no obvious JIA characteristic expression was found in the imaging, but it was consistent with the imaging performance reported by PPRD. As a result, a whole exome sequencing test for genetic diseases was performed. The findings revealed two mutations within the CCN6 gene, which included previously unreported PPRD-related mutation sites. These mutations were identified as follows: NM_003880.4 (CCN6) c.8T>C, classified with partial pathogenicity as VUS (variant of uncertain significance), and NM_003880.4 (CCN6) c.624dup, classified with suspected pathogenicity (**Figure 5**). This variation may be helpful to explain the clinic. Parents refused to take the test, so they were not evaluated.

The PPRD diagnosis of the patient was clear based on the patient’s symptoms, auxiliary examination, gene, and other results. Because PPRD does not an immune disease, we are giving it to him NSAIDs, prevention and treatment of osteoporosis, and traditional Chinese medicine. He is on follow-up, and there has been a marked reduction in the pain, although the deformity persists.

## 3 Discussion and conclusion

We report a novel case of PPRD with involving a novel CCN6 mutation and sacroiliac and hip arthritis, including bone marrow edema and joint effusion, which contradicts the previous assumption that PPRD is a noninflammatory disease.

The gene of c.802T>C mutations has been detected in the genes of Indian patients with PPRD (5), Chinese patients with epilepsy (6), and Czech patients with diabetes (7). The gene of c.624dup mutations has never been reported. This is the first report of c. 802T>C and c.624dup mutations in patients with PPRD in our country.

Previous reports indicate that patients with PPRD typically do not exhibit the characteristic bone erosion or bone marrow edema seen in JIA. However, the patient in this case presented with typical hip arthritis, along with bone marrow edema. This uncommon manifestation contributed to the misdiagnosis of JIA. These results suggest that PPRD patients have local inflammatory changes, such as bone marrow edema, which may be different from local joint inflammatory changes seen in the Enthesitis of SpA and the synovitis of JIA. In addition to that, heterozygous COL2A1 variants cause a wide spectrum of skeletal dysplasia-termed type II collagenopathies, which include a wide variety of skeletal dysplasia ranging from lethal disorders, such as achondrogenesis, type II or hypochondrogenesis (MIM#200610), to adult early onset osteoarthritis at the milder end of the spectrum (8). Spondyloepiphyseal dysplasia type Stanescu (SED-S, MIM 616583) is also the frequent forms of type II collagenopathies, characterized by COL2A1 gene mutation. One of the main differential diagnoses of SED-S is PPRD, which has overlapping clinical and radiologic manifestations of these two entities overlap (9). Both of these disorders require genetic testing to distinguish them from PPRD. Notably, some experiments have shown that WISP3, encoded by a gene mutated in PPRD, is located in the intermediate region of chondrocytes and regulates the expression of type II collagen (10). These data link SED-S to PPRD and may explain the overlapping phenotypes of the two entities.

Through searching the web of science database, a total of 55 cases of PPRD were reported, having flattened bones in the spine platyspondyly for 7%, abnormally shaped beaked for 32% (11–16), and bilateral symmetric bony enlargements of the interphalangeal joints for 100%.

The diagnosis of PPRD is specific imaging findings revealed spine platyspondyly and shaped beaked, osteoporosis, bilateral symmetric bony enlargements of the interphalangeal joints, and the characteristic pathogenic gene in CCN6. Therefore, early detection and diagnosis of PPRD can avoid unnecessary anti-inflammatory and immunosuppressive treatment, and it is very important to choose anti-osteoporosis treatment as early as possible, or choose corrective surgery or joint replacement surgery to help alleviate pain and disability related to PPRD. Although we can distinguish PPRD from SpA and JIA on typical spinal alterations and genetic tests, more cases and experiments are needed to demonstrate the relationship between specific clinical manifestations of PPRD and CCN6 gene variants, and to demonstrate the specific relationship between PPRD and SpA, JIA, and COL2A1 mutation.

## Data Availability

The data presented in the study are deposited in the Gene Bank repository, accession number SAMN44260485.
